# D-alanylation of lipoteichoic acids inhibitor provides anti-virulence and anti-resistance effects against methicillin-resistant *Staphylococcus epidermidis*

**DOI:** 10.1128/aac.01822-24

**Published:** 2025-03-21

**Authors:** Alexandre Mahé, Nicolas Verneuil, Delphine Coupri, Axel Hartke, Vincent Cattoir, Isabelle Rincé, Sabrina Gueulle, Xiao Feng, Thierry Lequeux, Emmanuel Pfund, Aurélie Budin-Verneuil

**Affiliations:** 1 CBSA UR 4312, Université de Caen Normandie 27003 , Caen, Normandy, France; 2 Department of Clinical Microbiology and National Reference Center for Enterococci, University Hospital of Rennes 27079 , Rennes, Brittany, France; 3 INSERM Unit U1230, University of Rennes, Rennes, Brittany, France; 4 Laboratoire de Chimie Moléculaire et Thioorganique LCMT UMR 6507, ENSICAEN, UNICAEN, CNRS, Normandie Université 357634 , Caen, Normandy, France; The Peter Doherty Institute for Infection and Immunity, Melbourne, Australia

**Keywords:** *Staphylococcus epidermidis*, methicillin-resistance, antibiotic resistance, D-alanylation, beta-lactams, teichoic acids, anti-virulence, anti-biofilm, D-alanylation inhibition, adjuvant

## Abstract

Methicillin-resistant *Staphylococcus epidermidis* (MRSE) is an emerging multidrug-resistant pathogen responsible for numerous healthcare-associated infections. Most of them are resistant to all classes of antibiotics and thus lead to therapeutic impasse. For this reason, identifying new targets and characterizing new drugs are essential. We recently showed that methicillin-resistant *Staphylococcus aureus* strains deficient in D-alanylation of teichoic acids (TAs) lost resistance to various β-lactams. Here we explore if D-alanylation of TAs might be a druggable target to overcome β-lactam resistance of MRSE using a competitive DltA inhibitor. The binding affinity of a DltA inhibitor with the purified DltA protein was monitored by determining the half maximal inhibitory concentration (IC_50_). The efficiency of D-alanylation inhibition was determined by quantifying the ester-linked D-alanine content of purified TAs. Minimal inhibition concentrations (MICs) and bactericidal effects of several β-lactams were monitored in the absence or presence of the inhibitor against a panel of clinical MRSE isolates. Finally, the ability of inhibition of D-alanylation (i) to rescue MRSE-infected larvae of *Galleria mellonella* and (ii) to prevent or eradicate *S. epidermidis* biofilms was evaluated. The DltA inhibitor showed IC_50_ in the low µM range, drastically reduced the D-alanine esters content of TAs and re-sensitized MRSE to β-lactams. The most effective treatment was the DltA inhibitor/imipenem combination. Finally, inhibition of D-alanylation significantly reduced the virulence of MRSE in the *G. mellonella* infection model and strongly reduced the ability of *S. epidermidis* to form biofilms. All together, our results show the promising nature of the D-alanylation of TAs as a therapeutic target to fight against MRSE infections.

## INTRODUCTION

Among species of coagulase-negative staphylococci (CoNS), *Staphylococcus epidermidis* is a major representative of the human skin microbiota (1). This commensal bacterium is recognized for its beneficial effects on health, such as maintaining skin homeostasis and skin barrier protection against pathogenic bacteria, including *Staphylococcus aureus* (1–3). However, many studies have also reported *S. epidermidis* as an emerging opportunistic pathogen responsible for numerous hospital-acquired infections (HAIs), especially device- and biofilm-related infections (4–6). Recently, two studies conducted in the United States between 2015 and 2019 showed that CoNS ranked 6th and 3rd among HAI pathogens in adults and children, respectively, with *S. epidermidis* being responsible for 47.4% and 58.3% of these CoNS infections (7, 8). Besides, there is an increase in the prevalence of methicillin-resistant *S. epidermidis* (MRSE) that carry the staphylococcal cassette chromosome *mec* (SSC*mec*) harboring the *mecA* gene encoding the β-lactam low affinity penicillin-binding protein (PBP), named PBP-2a. In addition, most of these isolates are resistant to other classes of antibiotics, such as aminoglycosides, fluoroquinolones, macrolides-lincosamides-streptogramins, tetracyclines, rifampicin and cotrimoxazole (9–11). Therefore, these infections caused by CoNS are often difficult to treat with currently-approved antibiotic therapies.

A strategy to fight multidrug-resistant (MDR) bacteria is to identify molecules that might be used as adjuvants to restore therapeutic effectiveness of antibiotics against which the pathogen has become resistant. In this context, the D-alanylation of teichoic acids (TAs) represents a very promising target (12, 13). Teichoic acids, comprising both wall teichoic acids (WTAs) and lipoteichoic acids (LTAs), are anionic glycopolymers present in the cell wall of most Gram-positive bacteria. The negative charge of TAs can be modulated by tagging D-alanine residues through a D-alanylation mechanism, which requires the involvement of five essential proteins (all encoded by the *dltXABCD* operon) (14, 15). In 2005, May and collaborators showed that an inhibitor targeting the D-alanyl carrier protein ligase DltA increased the susceptibility of *Bacillus subtilis* to vancomycin (16). More recently, we have shown that clinical isolates of enterococci and methicillin-resistant *S. aureus* (MRSA) were sensitized by treatments combining the same DltA inhibitor with β-lactam antibiotics. Moreover, these treatments significantly reduced the virulence of those pathogens, including biofilm formation in MRSA (17, 18).

In this study, we checked *in vitro* the DltA inhibitor efficiency against *S. epidermidis* DltA, then we analyzed the susceptibility of a panel of clinical *S. epidermidis* isolates by combining the DltA inhibitor with various β-lactams. First, we demonstrated that the strains were more susceptible to β-lactams in the presence of the DltA inhibitor, and that this susceptibility was correlated with a decrease in cell wall D-alanylation. Second, we showed that these treatments strongly reduced the ability of *S. epidermidis* to form biofilms. Finally, we evidenced using the *Galleria mellonella* infection model that the inhibitor used alone significantly affected the virulence of MRSE isolates. All together, these results confirm that the inhibition of D-alanylation of TAs is a promising therapeutic strategy to fight against MDR Gram-positive pathogenic bacteria.

## MATERIALS AND METHODS

### Comparison of the DltA protein structures

The structures of the DltA protein from *B. subtilis* (*Bsu*DltA, PDB ID: 3E7W, 508 amino acids (aa)) and *S. epidermidis* (*Se*DltA, AlphaFold ID: AF-Q5HQN0-F1-v4, 485 aa) were compared using the ‘align’ command in PyMOL (PyMOL Molecular Graphics System, Version 3.0 Schrödinger, LLC), with the ‘cycles’ argument set to values ranging from 0 to 5.

### Bacterial strains, media, and reagents

Bacterial strains used in this study are listed in Table 1. S. *epidermidis* cultures were grown in Brain-Heart Infusion (BHI) (Biokar Diagnostics, France) with shaking (120 rpm). *E. coli* strains were grown in Luria-Bertani (LB) broth (10 g/L tryptone, 5 g/L yeast extract, 5 g/L NaCl, pH 7.1) (Bertani, 1951) with shaking (120 rpm) for plasmid construction or in Terrific broth (20 g/L tryptone, 24 g/L yeast extract, 4 mL glycerol, 0.17 M KH_2_PO_4_, 0.72 M K_2_HPO_4_) (19) with shaking (120 rpm) for protein overexpression. Imipenem (IPM), erythromycin (ERY), kanamycin (KAN) and cefoxitin (FOX) were supplied by Sigma-Aldrich (MO, USA) and oxacillin (OXA) was purchased from VWR (PA, USA). DltA inhibitor {5′-O-[N-(D-alanyl)-sulfamoyl]-adenosine} was synthesized as described by May et al. (16) and solubilized at the concentration of 10 mM in sterilized pure water.

**TABLE 1:** Strains and plasmids used in this study^*a*^

Strains	Origin / Reference	Communicated resistances / Features
*S. epidermidis*		
MRSE 1^*b*^	Deep pus	ERY, FA, KAN, OFX, OXA, RIF,
MRSE 2^*b*^	Joint fluid	ERY, FA, FOX, KAN, MOX, OXA,
MRSE 4^*b*^	Intra-abdominal fluid	FA, FOX, MOX, OFX, OXA,
MRSE 5^*b*^	Endotracheal aspirate	ERY, FA, GEN, KAN, OXA, RIF, TEC
MRSE 6^*b*^	Deep pus	ERY, FA, GEN, KAN, OFX, OXA,
MRSE 7^*b*^	Biopsy	ERY, FA, FOX, GEN, KAN, MOX, OFX, OXA,
MRSE 8^*b*^	Deep pus	ERY, FA, GEN, KAN, OFX, OXA,
MRSE 9^*b*^	Catheter	CLI, ERY, FA, FOX, GEN, KAN, LVX, MOX, MXF, OFX, OXA, RIF
MRSE 10^*b*^	Bronchial aspirate	CLI, CPT, ERY, FOX, GEN, KAN, LZD, LVX, MOX, MXF, OFX, OXA, RIF, TEC
MRSE 11^*b*^	Blood culture	ERY, FA, FOX, GEN, KAN, LVX, MOX, MXF, OFX, OXA, RIF, TEC
MRSE 12^*b*^	Blood culture	CLI, ERY, FA, FOX, GEN, KAN, LVX, MOX, MXF, OFX, OXA, TEC
MRSE 13^*b*^	Joint fluid	FA, FOX, GEN, KAN, LVX, MOX, MXF, OFX, OXA, RIF
MRSE 14^*b*^	Catheter	ERY, FA, FOX, GEN, KAN, LVX, MOX, MXF, OFX, OXA
MRSE 15^*b*^	Blood culture	ERY, FA, FOX, GEN, KAN, LVX, MOX, MXF, OFX, OXA
MRSE 16^*b*^	Unknown	FA, FOF, FOX, GEN, KAN, LVX, MOX, MXF, OFX, OXA
MRSE 18^*c*^	Blood culture	OXA, FOX
MRSE 19^*c*^	Blood culture	OXA, FOX
MRSE 20^*c*^	Osteoarticular infection	OXA, FOX
MRSE 21^*c*^	Blood culture	OXA, FOX
MRSE 22^*c*^	Blood culture	OXA, FOX
MRSE 23^*c*^	Catheter	OXA, FOX
MRSE 24^*c*^	Blood culture	OXA, FOX
MRSE 25^*c*^	Skin infection	OXA, FOX
MRSE 26^*c*^	Blood culture	OXA, FOX
MSSE 1^*c*^	Blood culture	MSSE strain
ATCC 14990	Nasal swab	Reference strain
*E. coli*		
BL21(DE3)	(20, 21)	DE3 prophage, *lon*(-), *ompT*(-), *dcm*(-), *hsdS_B_* (r_B_^-^ m_B_^-^)
BL21 pET-dltAR6	This study	BL21(DE3) carrying DltA from MRSE 6 overexpression vector
BL21 pET-dltCR6	This study	BL21(DE3) carrying DltC from MRSE 6 overexpression vector
Plasmids		
pET29b(+)	Novagen	ori pBR322, KAN, *lacI*, His-Tag in C-ter, *lacO*, T7 promoter
pET-dltAR6	This study	pET29b(+) carrying MRSE 6 *dltA* gene, His-Tag in C-ter
pET-dltCR6	This study	pET29b(+) carrying MRSE 6 *dltC* gene, His-Tag in C-ter

^*a*^ CLI : Clindamycin ; ERY : Erythromycin ; FA : fusidic acid ; CPT : Ceftaroline ; FOF : Fosfomycin ; FOX : Cefoxitin ; GEN : Gentamicin ; KAN : Kanamycin ; LZD : Linezolid ; LVX : Levofloxacin ; MOX : Moxalactam ; MXF : Moxifloxacin ; OFX : Ofloxacin ; OXA : Oxacillin ; RIF : Rifampicin ; TEC : Teicoplanin.

^*b*^ Clinical isolates from UHC of Caen.

^*c*^ Clinical isolates from UHC of Rennes. C-ter: C-terminal end of the protein.

### Growth kinetics, MIC determination, and bacterial survival

Colonies from BHI agar plates were suspended in saline solution (0.9% NaCl) and adjusted to an OD_600_ of 0.5. For growth kinetics determination, 96-Well microplates (Starlab, France) containing 200  µL of fresh BHI medium were inoculated with bacterial suspensions to an OD_600_ of 0.02 with a wide range of DltA inhibitor (from 0 to 2  mM). Plates were incubated at 37°C with shaking (orbital amplitude of 3  mm) in a microplate reader (Infinite M Nano, Tecan). OD_600nm_ was measured every 10  min for 24  h. Determination of MIC/MBC of antibiotics and bacterial survival assays were performed as previously described (17, 18) using BHI medium. The DltA inhibitor was diluted in pure water and added to the growth medium to a final concentration of 0, 0.1, 0.25, 0.5, 0.75, or 1 mM.

### Quantification of D-alanyl ester in the bacterial cell wall

Ester-linked D-alanines were quantified as described previously (22), with the following modifications: cultures were grown in 5 mL of BHI supplemented with DltA inhibitor to a final concentration of 0.05, 0.1, 0.25, 0.5, 0.75 or 1 mM for 16 h at 37°C under shaking (120 rpm). Briefly, the quantification is based on the oxidation of a chromogen by H_2_O_2_ generated when free D-alanines are oxidized by a D-amino acid oxidase. Three independent experiments with duplicate samples were performed.

### *G. mellonella* infection model and in host efficacy of antibiotic/inhibitor combination

The experiments to determine virulence and effects of treatments were realized using larvae of *G. mellonella* as previously described (18). Strains of *S. epidermidis* were cultured in BHI broth and then adjusted to reach an infection dose of around 5 × 10^6^ CFU/larva. Imipenem (0.6 mg/kg) and DltA inhibitor (24.2, 36.4 or 48.5 mg/kg) were administered 2 h post-infection. Bacterial inoculum size was checked by plate counting on BHI agar. For colonization assays, the larvae were infected with around 10^3^ CFU of MRSE 11 and treated as previously with DltA inhibitor. In the host, bacterial load at times 0 and 24 h post-infection was evaluated by plating the crushed larvae on BHI agar. MRSE 11 isolate was distinguished from the commensal flora by plating on BHI agar containing erythromycin at 75 µg/mL.

### Biofilm inhibition and eradication

The biofilm inhibition (BI) and eradication (BE) experiments were performed as described by Coupri et al. (18) with the following modifications. Biofilms were not air-dried a second time after crystal violet staining. The optical density at 570 nm was assessed with an Infinite M Nano microplate reader (Tecan, Männedorf, Switzerland). To count viable intra-biofilm cells, the same protocol as BE was performed, but microplates were treated 5 min at 40 kHz at room temperature with a M1800H bath sonicator (Brookfield, CT, USA) instead of being colored. Dispersed biofilm was then plate-counted on BHI agar.

### Construction of DltA- and DltC-overexpressing plasmids

Primers used in this study are shown in Table S1. PCRs were carried out with Q5 polymerase (New England Biolabs, MA, USA). Thermal programs were achieved by a Mastercycler Nexus gradient thermal cycler (Eppendorf, Germany). Chromosomal DNA of *S. epidermidis* MRSE 26 was extracted using the Nucleospin Microbial DNA kit (Macherey-Nagel, France) following the manufacturer’s instructions. The *dltA* and *dltC* genes were then amplified using primer couples F_SE_pET*dltA*/R_SE_pET*dltA* and F_SE_pET*dltC*/R_SE_pET*dltC,* respectively. In parallel, the expression vector pET29b(+) was amplified using primers OCG232 and OCG233. Remaining circular copies were restricted with a *Dpn*I (Promega, WI, USA) treatment. Gene amplicons and linearized vectors were transformed in *E. coli* BL21 for *in vivo* homologous recombination (20).

### Chemically competent cells preparation and transformation

Buffers used in this study are shown in Table S2. *E. coli* BL21 (21) was grown overnight in 10 mL of LB with shaking (120 rpm), then diluted 100-fold in fresh LB and re-incubated. When the OD_600_ reached 0.5, the culture was aliquoted into 1.5 mL Eppendorf tubes, centrifuged (1000 x g, 10 min) and resuspended in 90 µL of TSS for storage at −80°C. When needed, an aliquot was gently thawed on ice and 10 µL of KCM 5X was added. Linear pET29b(+) and gene amplicon were mixed in a 6:4 ratio and 10 µL were added to competent cells for a first incubation at 4°C (30 min), then a second incubation at 42°C (45 s). Five hundred µL of LB broth was added and incubated 1 h at 37°C. After recovery, cells were plated on LB agar supplemented with 25 µg/mL of kanamycin. Transformation of resulting colonies and *in-vivo* recombination were checked by colony PCR amplification using OCG240 and OCG241 primers.

### DltA and DltC overexpression and purification

In a baffled Erlenmeyer flask, 250 mL of terrific broth was inoculated at a starting OD_600_ of 0.1 with an overnight (ON) culture of *E. coli* BL21 carrying the over-expression vector, then grown at 37°C with orbital shaking (160 rpm) until the OD_600_ reached 0.5. IPTG was added at a final concentration of 0.5 mM during 3 h at 37°C with orbital shaking (160 rpm). The obtained culture was washed twice with 100 mL, then 20 mL of purification buffer (Tp; Table S2) and centrifuged (4500 rpm, 15 min, 4°C). The pellet was suspended in Tp supplemented with 1 mg/mL of lysozyme and incubated for 30 min at 4°C, then ultrasonicated with a Sonic Dismembrator (Fisher Scientific, Waltham, USA). Supernatant was eluted using a 5 mL HisTrap FF Crude (GE Healthcare, Chicago, USA) set up on a ÄKTA Start system (Cytiva, Munzinger, Germany). Impurities were eluted with 15 mL (2.5 mL/min) of Tp containing 50 mM of imidazole, then the protein of interest was eluted with a gradient of 65 mL of imidazole from 60 mM to 500 mM (2.5 mL/min). Fractions of interest were pooled on a 15 mL Vivaspin Turbo RC 50 kDa MWCO column (Sartorius Stedim Biotech, Göttingen, Germany) for concentration, then desalinated on a PD-10 column following manufacturer recommendation (GE Healthcare, Chicago, USA) with EB-3 buffer (Table S2) as eluent. Eluate was concentrated a second time with a 6 mL Vivaspin 30 kDa MWCO column (Sartorius Stedim Biotech, Göttingen, Germany) and stored at −80°C. Purification was checked on 12.5% acrylamide SDS-PAGE gel using Coomassie blue staining and a PageRuler Prestained Protein Ladder (Fisher Scientific, Waltham, USA). Purified DltA and DltC proteins were verified by MS/MS sequencing.

### Determination of the half-maximal inhibitory concentration (IC_50_)

The DltA-catalyzed ATP-hydrolysis reaction was monitored *in vitro via* PPi release (DltA +D-alanine +ATP → DltA-DAla-AMP +PPi) using a coupled-enzyme assay. The purified DltA was pretreated with DltC purified from *E. coli* to clear D-Ala-AMP already present in the enzyme preparation after expression in *E. coli*. Holo-DltC is the natural protein partner of DltA, and DltC purified from *E. coli* BL21 (DE3) contained 51% of this active form of DltC as determined by HPLC analysis. DltA was pretreated with DltC (ratio 1:1.2), incubated 30 min at 37°C and 30 min at 4°C. Three successive washes were realized on Amicon Ultra-2 Centrifugal Filter Unit – Ultracel 30 membrane 30 kDa MWCO (Merck, Darmstadt, Germany) with elution buffer EB at 4°C. The components of the coupled enzyme assay included: 1.5 µM pre-treated DltA, 20 µM of ATP, and 14 µM of D-alanine. The half-maximal inhibitory concentration (IC_50_) of potential DltA inhibitors was determined by the addition of different concentrations of the synthesized molecules. PPi release was determined using the PiPer – Pyrophosphate assay kit (Invitrogen, Waltham, MA, USA) according to the recommendation of the supplier. Reactions were carried out in 96-well Chimney Style, non-binding Microplates with μClear Film Bottom (Greiner Bio-one, Frickenhausen, Germany). Plates were incubated at 37°C for 5 h and read in FlexStation 3 Multi-Mode plate reader (Molecular Devices, Silicon Valley, CA, USA), with λ_excitation_ of 530 nm and λ_emission_ of 590 nm. Experiments were conducted in sextuplicate, and the IC_50_ was determined by linear regression of the curve.

## RESULTS

### DltA proteins from *B. subtilis* and *S. epidermidis* share high structural similarity

**Fig 1:**
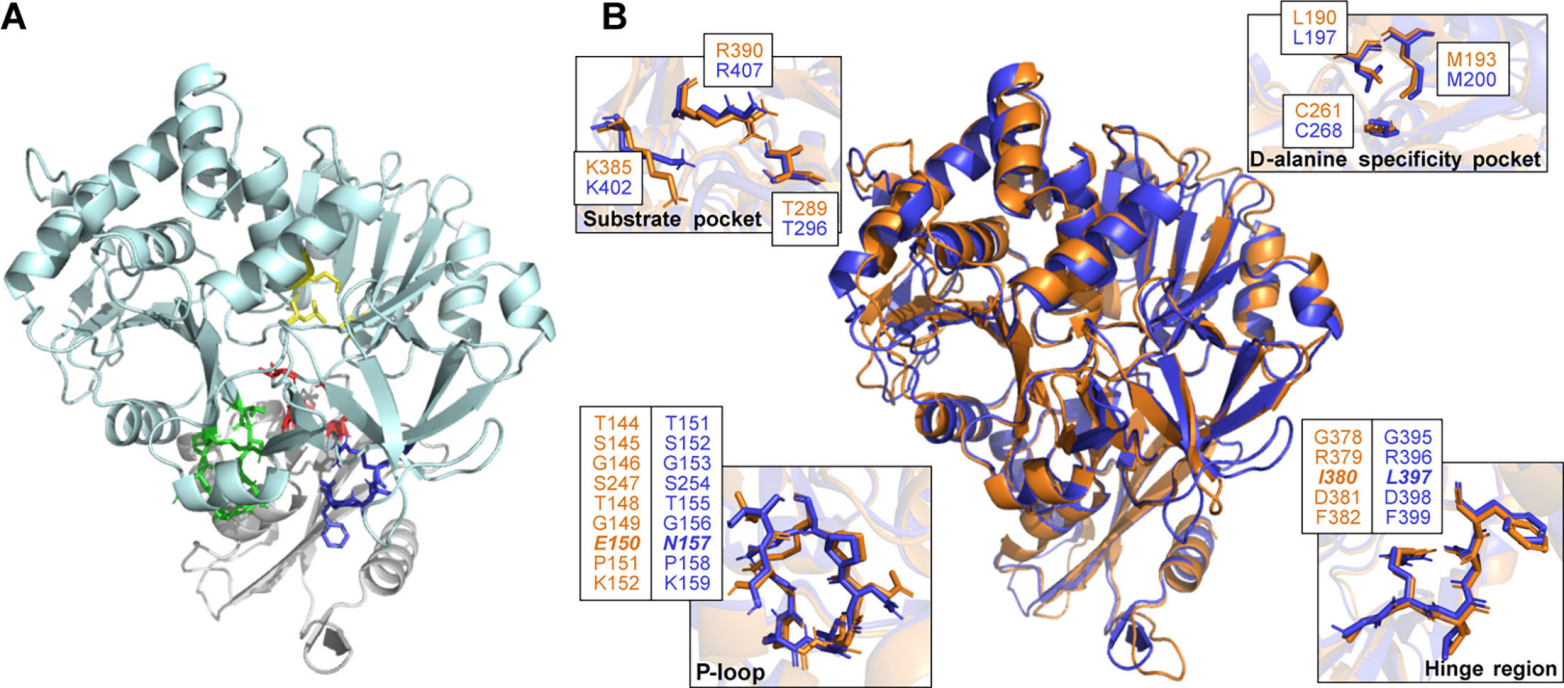
Functional structure of DltA. (A) Structure of DltA from *B. subtilis* MR168 in the thiolation conformation (adapted from Yonus et al. [23]) with the large N-terminal subunit (light cyan) containing the D-alanine specificity pocket (yellow) and the small C-terminal subunit (light grey) containing the P-loop (green) and the hinge region (blue). The substrate-binding pocket (red), that interacts with ATP and Mg^2+^, shares aa between both large and small subunits. (B) Structural superposition of DltA from *B. subtilis* MR168 (blue) and the structure of DltA from *S. epidermidis* ATCC 35984 predicted with AlphaFold (orange). The aa that constitute each region are indicated in the boxes. Residues in bold italic indicate the aa differing between the two structures.

The structure of *Bsu*DltA has been previously deduced by Yonus et al. (23). It consists of two subunits, the large N-terminal lobe and the small C-terminal lobe, linked together by a flexible four-residue hinge loop (Fig. 1A). This hinge allows an open/closed conformation switch once the substrates (ATP/Mg^2+^ on one side and D-alanine on the other) are bound to the substrate-binding pocket and the D-alanine specificity pocket, respectively, bringing into proximity the different partners in the active site. Then, the adenylation of D-alanine and the release of pyrophosphate trigger the rotation of the small lobe into a thiolation conformation. In this state, DltA would transfer the activated D-alanyl moiety to the phosphopantheinyl prosthetic group of DltC. The P-loop is also contributing to the conformational changes by interacting with the triphosphate moiety of ATP and some residues of the small region. Since the DltA inhibitor used in this study is a nonhydrolysable analogue of the D-alanyl adenylate reaction intermediate, it is assumed that this molecule is a competitive inhibitor of the enzyme, which is in agreement with the shape of the Dixon plot obtained by May et al. (16). Therefore, we compared the structures of *Bsu*DltA and *Se*DltA to ensure that the inhibitor could also work on *Se*DltA (Fig. 1B). Depending on the number of cycles of refinement (from 1 to 5), the superposition of both structures indicated an RMSD ranging from 2.22 Å (480 aa aligned) to 0.718 Å (377 aa aligned), indicating that the general structure is well conserved. Regarding the substrate and D-alanine specificity pockets, both structures are perfectly homologous and present the same spatial distribution. The P-loop and the hinge region differ by one amino acid each (*Se*DltA-E150 instead of *Bsu*DltA-N157 and *Se*DltA-I380 instead of *Bsu*DltA-L397, respectively), but their overall three-dimensional structures are well conserved.

### DltA inhibitor targets *S. epidermidis* DltA protein *in vitro*

The DltA protein of *S. epidermidis* was heterologously expressed in *E. coli* and purified (Fig. S1). K_m_ and V_max_ of *Se*DltA were determined using the *renz* R package (24), with values of 5.43 ± 2.03 µM and 279 ± 17.22 RFU.h^−1^ respectively (see Fig. S2). This K_m_ value is in the same order as previously published for *B. subtilis*, *Bacillus cereus,* and *S. aureus* (16, 25, 26). Unfortunately, we could not determine the K_i_ because the DltA inhibitor previously described by May et al. (16) interfered with the coupled enzyme assay used to measure DltA activity. However, we were able to measure the IC_50_ value using the coupled enzymatic assay since 10-fold lower concentrations of the inhibitor are used compared to the K_i_ analyses. From six replicates, an IC50 of 2.43 ± 0.33 (mean ± SD) µM was determined. This showed that the inhibitor has a good affinity for the *S. epidermidis* protein *in vitro*.

### DltA inhibitor reduces MICs of β-lactams against MRSE

**Fig 2:**
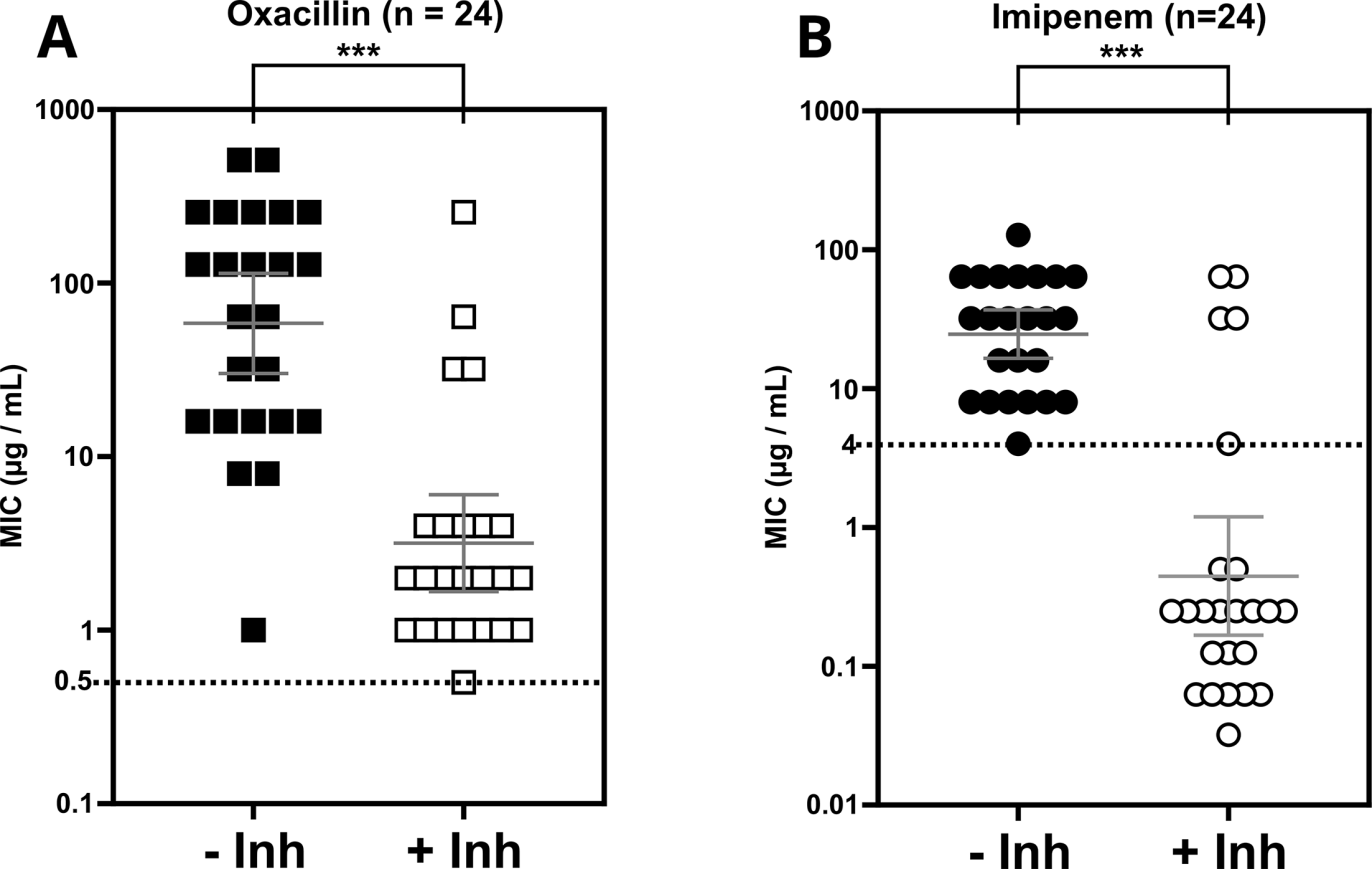
Effect of DltA inhibitor on MICs of oxacillin and imipenem against MRSE clinical isolates. MICs of oxacillin (A; squares) and imipenem (B; circles) were assessed after 24 h of incubation in BHI medium in the absence (full) or presence (empty) of DltA inhibitor (Inh) at 1 mM. Each dot represents the most frequently observed MIC among at least three biological replicates per clinical isolate. N-values (n) give the number of strains assessed for each antibiotic. Gray bars correspond to the geometric mean and the 95% CI. Dotted lines show the resistance thresholds according to 2020 CLSI guidelines. *** *P*-value < 0,0005 [one-tailed Wilcoxon matched-pairs signed rank test, realized on the “GraphPad Prism 8.0.1” software (Boston, Massachusetts USA, www.graphpad.com)]. Values of MICs and MBCs of antibiotics against each MRSE strain are summarized in Table S3.

With the aim of finding a combinatory treatment able to re-sensitize MRSE to β-lactam antibiotics, MICs of OXA, FOX, and IPM against a panel of 24 MRSE clinical isolates were determined in the absence or presence of 1 mM of DltA inhibitor in BHI broth. The strains showed variable profiles of resistance to the tested β-lactams, with MICs ranging from 4 to 128 µg/mL for IPM, from 16 to 512 µg/mL for FOX, and from <2 to 512 µg/mL for OXA (Fig. 2; Table S3).

**Fig 3:**
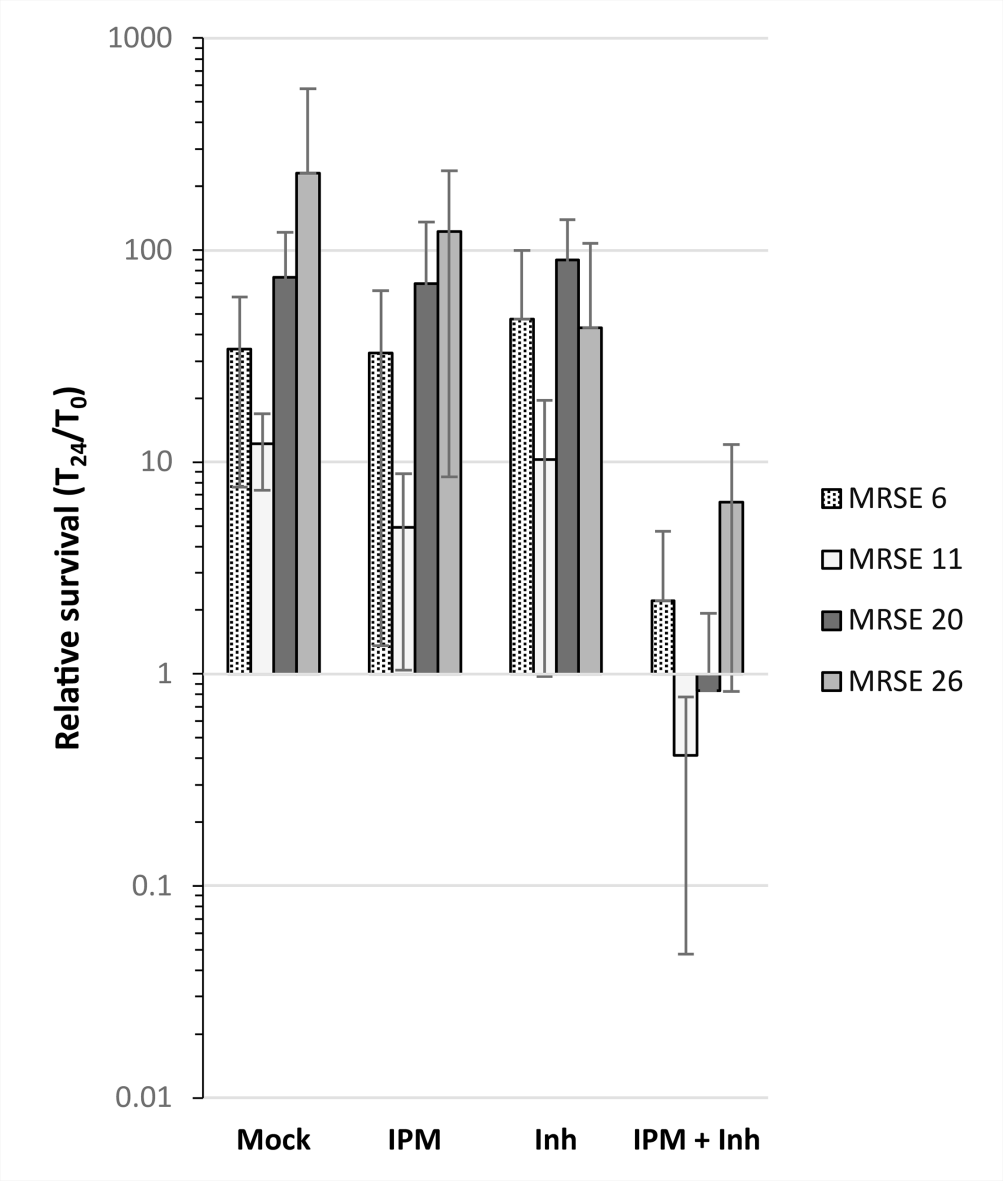
Relative survivals of MRSE-clinical isolates in the presence of IPM/DltA inhibitor combination reveal a bacteriostatic effect. Early-log phase cultures of *S. epidermidis* MRSE 6 (dotted), MRSE 11 (clear), MRSE 20 (black) and MRSE 26 (grey) were incubated at 37°C at time 0 (T_0_) in absence (Mock) or presence of 1 µg/mL of IPM, 1 mM of DltA inhibitor (Inh) or both (IPM + Inh). After 24 h (T_24_), the survival was determined by plate counting and the relative survival (T_24_/T_0_) was calculated. Results are represented as the mean ± SD survival from three biological replicates.

Globally, the DltA inhibitor significantly decreased MICs of OXA and IPM for most MRSE isolates (Fig. 2; Table S3). The molecule conferred an 8-fold decrease of OXA MICs for 87.5% of tested strains. Although this decrease was significant, MICs were still above the susceptibility breakpoint recommended by CLSI in 2020 (i.e. ≤0.25 µg/mL). Regarding the MICs of FOX in the presence of the inhibitor, a turbidity gradient across the FOX concentration range was often observed, making the first clear well difficult to distinguish visually. To note, no growth default was observed for concentrations of the DltA inhibitor up to 1 mM (Fig. S3). For this reason, the MIC for FOX in the presence of the inhibitor could be unambiguously determined only for seven isolates. For five of them (71.4%), the DltA inhibitor conferred a fourfold decrease in FOX MICs (Fig. S4). Regarding IPM, the addition of the DltA inhibitor reduced MICs between 8- and 256-fold for 83.3% of the tested isolates, and regardless of their degree of resistance. All these isolates were re-sensitized to imipenem by the action of the inhibitor in accordance with CLSI 2010 recommendations (Table S3). However, four isolates were resistant to the combinatorial treatment IPM/DltA inhibitor. Of note, OXA and IPM MBCs were also decreased at least fourfold for 75% of the tested strains. To determine whether a lower dose of DltA inhibitor reduced efficiently the MIC of IPM, a range from 0.1 to 1 mM of the drug was tested against four MRSE isolates with various levels of resistance. This showed that 0.5 mM of DltA inhibitor was the minimal concentration to decrease the MIC of IPM significantly for three strains, even though 1 mM was required to re-sensitize *S. epidermidis* below the IPM breakpoint (Table S4). We concluded that the DltA inhibitor efficiently potentiated IPM action against *S. epidermidis*. Since drugs are considered bactericidal if survival decreases by ≥3 log in 24 hours, the IPM/inhibitor combinatorial treatment, although a reduction of survival of approximately 1-log was observed for some strains, had rather a bacteriostatic effect (Fig. 3; Fig. S5). These results are in accordance with MBC measurement, since the MBC/MIC ratio is equal to or higher than 4 for 75% of tested strains (Table S3).

### DltA inhibitor decreases wall D-alanylation of *S. epidermidis*

**Fig 4:**
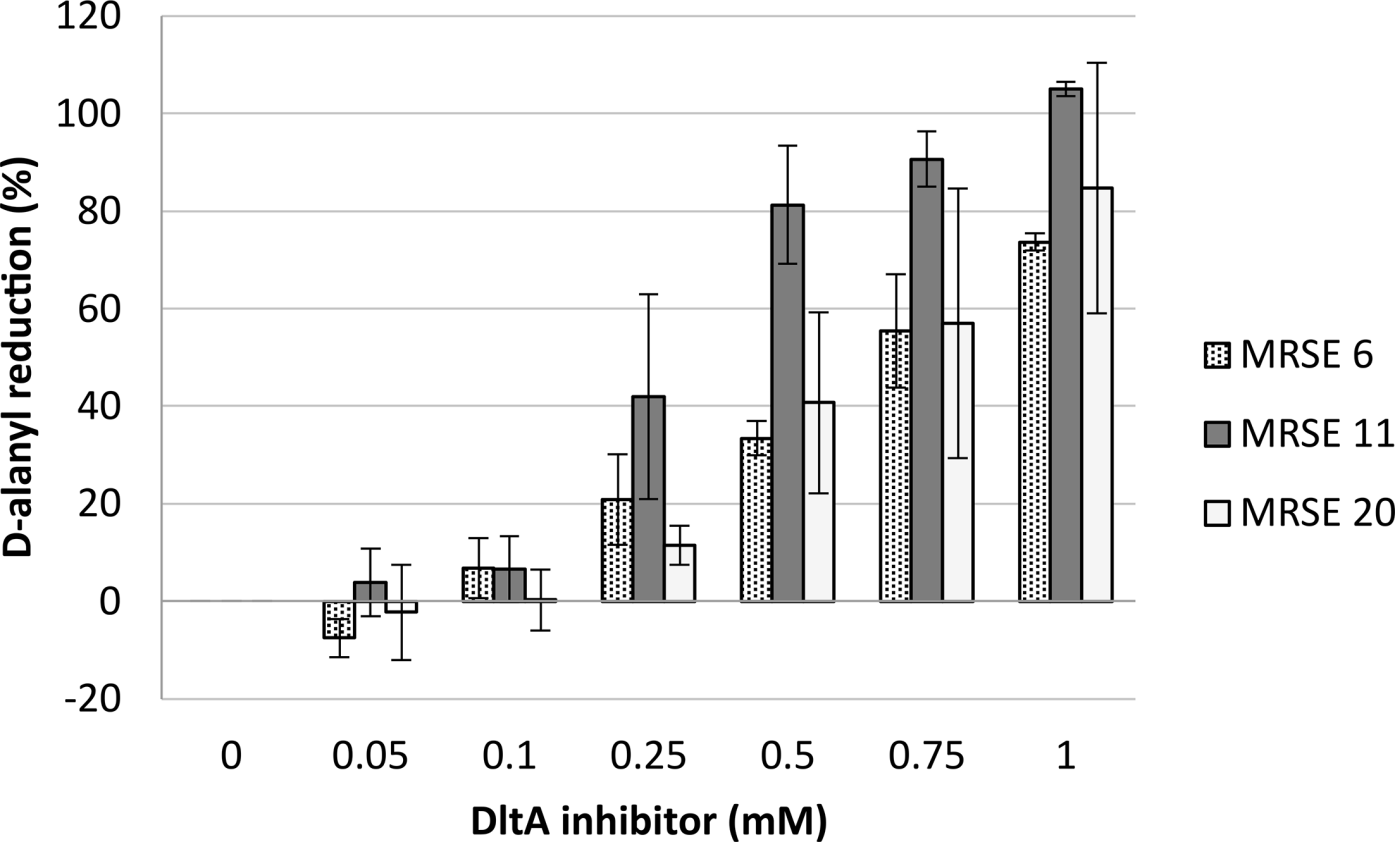
DltA inhibitor inhibits cell wall D-alanylation of *S. epidermidis* in a dose-dependent way. MRSE 6 (dotted), 11 (clear), and 20 (dark) strains were grown in BHI for 16 h with a range of concentration of DltA inhibitor (from 0.05 to 1 mM). Data are represented as the mean ± 95% CI, corresponding to the ratio of ester-linked D-alanine content lost when treated compared to untreated condition. Three biological replicates, each containing two technical replicates, were assessed.

To ensure that the inhibitor blocks D-alanylation *in vivo*, wall D-alanyl residues were quantified in MRSE 6, MRSE 11, and MRSE 20 strains in the presence of different concentrations of DltA inhibitor. This showed that the drug inhibited wall D-alanylation in a dose-dependent manner (Fig. 4). At 0.25 mM, the DltA inhibitor reduced the level of D-alanylation by 42%, 20% and 11.5% for MRSE 11, MRSE 6 and MRSE 20, respectively. This increasing inhibition correlates well with the increased susceptibility to IPM at these concentrations of the inhibitor (Fig. 4; Table S4). At 1 mM, inhibition was close to 100% for all tested strains (Fig. 4; Fig. S6).

### DltA inhibitor improves survival of *G. mellonella* infected with MRSE

**Fig 5:**
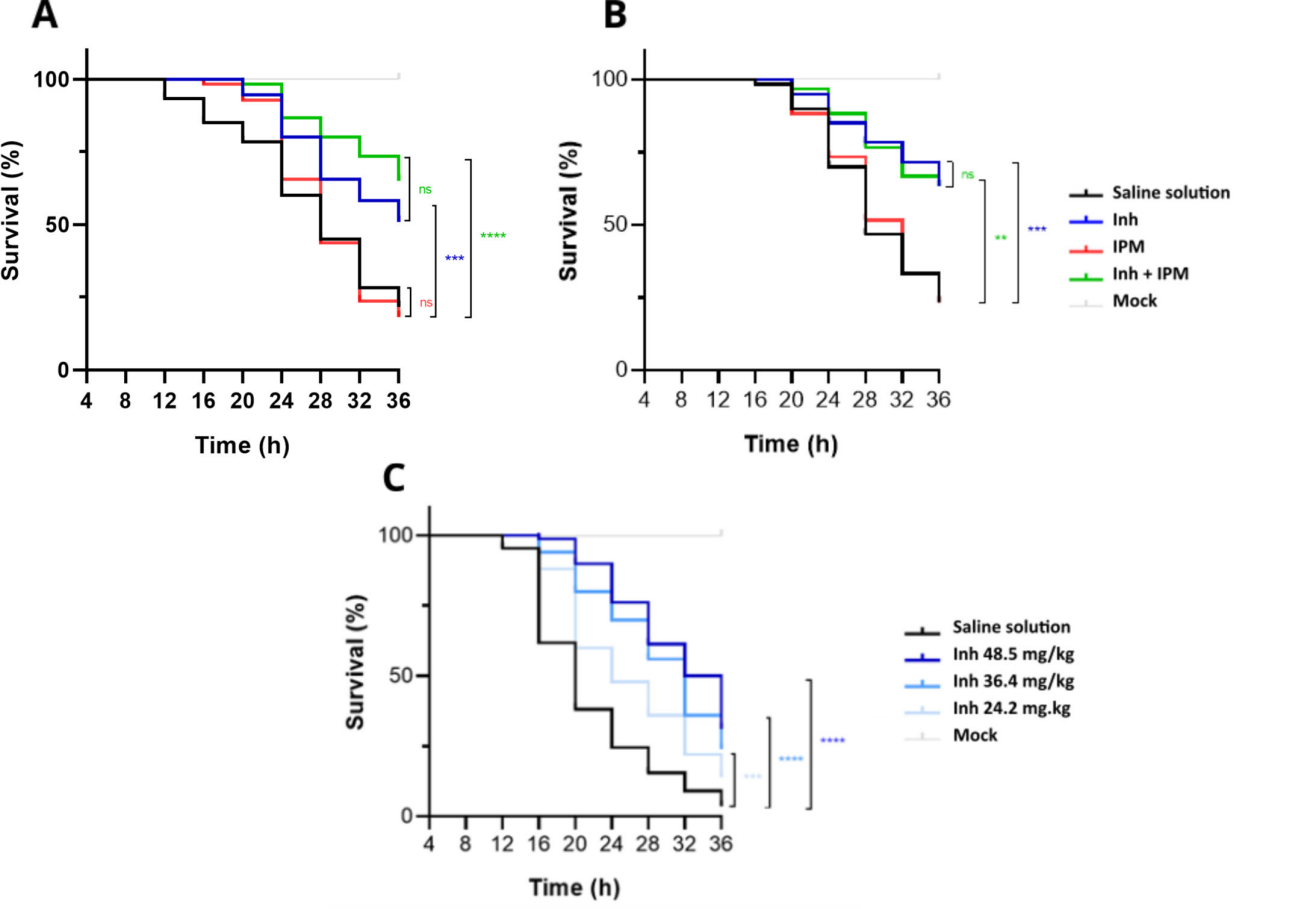
Inhibition of D-alanylation improves survival of *G. mellonella* infected by MRSE isolates. Six-week-old *G. mellonella* larvae were infected (or mock-infected) by a lethal dose of MRSE 11 (A), MRSE 20 (B) and treated 2 h post-infection with saline solution (black line), IPM (0.6 mg/kg; red line), DltA inhibitor (48.5 mg/kg; blue line) or IPM/DltA inhibitor combination (green line). The same protocol was followed with MRSE 10 (C) for which larvae were treated with a range of concentrations of DltA inhibitor (24.2, 36.4 or 48.5 mg/kg; gradient blue lines). Alive and dead larvae were counted every 4 h, from 8 to 36 h post-infection. At least 60 larvae per condition were treated. Data are presented on Kaplan-Meier curves, and log-rank statistical analysis was performed with GraphPad Prism 8.0 to compare with saline solution condition (GraphPad Softwares Inc. ns: not significant; **P-value < 0.004; ***P-value < 0.0004; ****P-value < 0.0001).

To assess the ability of the DltA inhibitor to improve *G. mellonella* survival in a MRSE infection model, larvae were infected with lethal doses of strains MRSE 11 (Fig. 5A) or MRSE 20 (Fig. 5B) and treated by injection of saline solution, IPM (0.6 mg/kg), DltA inhibitor (48.5 mg/kg) or IPM/DltA inhibitor combination at the same concentrations 2 h post-infection. Virulence of both strains was very similar with a final survival of approximately 25% after 36 h post-infection. Treatment with IPM alone had no effect on survival of the larvae. When treated with the DltA inhibitor alone, survival of the larvae infected with MRSE 11 (Fig. 5A) or MRSE 20 (Fig. 5B) significantly increased (*P*-value < 0.0004) by 30% and 35% after 36 h post-infection, respectively. Colonization assays, conducted on BHI plates containing erythromycin to suppress most of the commensal flora of the larvae, showed that this was not due to growth differences in the presence or absence of the inhibitor since in both conditions a similar (100-fold) increase in MRSE 11 CFUs in the larvae were observed after 24 h of infection (Fig. 5A; Fig. S7). These results therefore suggest an anti-*S*. *epidermidis* virulence activity of the drug. Unexpectedly, the combined treatment IPM/DltA inhibitor did not or only slightly improve *G. mellonella* survival compared to the inhibitor alone for strains MRSE 20 and MRSE 11, respectively (Fig. 5A and B). In this context, we wondered if the MRSE 10 strain, which is resistant to all β-lactam/DltA inhibitor combinations, would also be resistant to the anti-virulence effect of the inhibitor. Larvae were infected with the MRSE 10 strain and treated with the DltA inhibitor (24.2, 36.4, or 48.5 mg/kg) 2 h post-infection. This showed that survival of the larvae infected with MRSE 10 increased proportionally to the amount of DltA inhibitor, with a rise of 30% for the highest inhibitor concentration (Fig. 5C).

### Combinatorial treatment DltA inhibitor/IPM affects *S. epidermidis* biofilm formation

**Fig 6:**
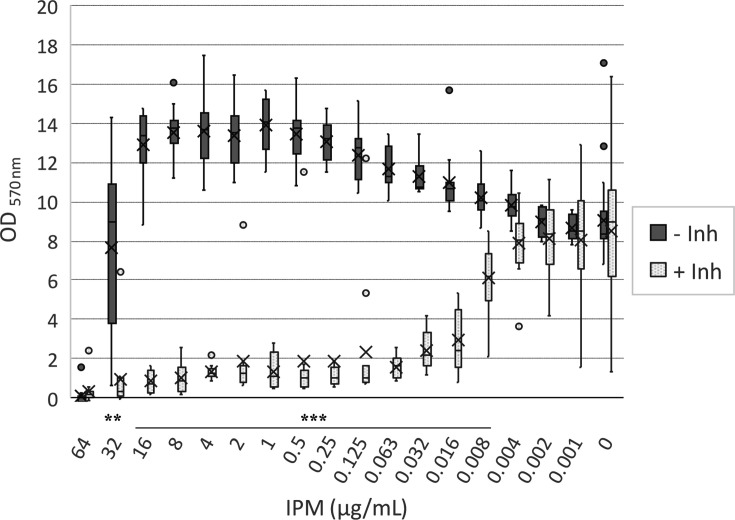
Anti-biofilm effect of DltA inhibitor combined with imipenem in *S. epidermidis*. Biofilm formation inhibition of imipenem in the absence (dotted) or presence (grey) of 0.5 mM of DltA inhibitor was assessed against MRSE 11 strain grown in BHI supplemented with 2% glucose for 24 h. Data are represented in box plots, providing the distribution of three biological replicates containing each four technical replicates as well as the means (cross), medians (horizontal bars) and outliers (circles). The values are corrected by subtracting the mean of negative controls (medium only). A two-tailed Welch’s *t*-test was performed for each concentration of IPM using GraphPad Prism 8.0 (GraphPad Software Inc. **: *P*-value = 0.0002; ***: *P*-value < 0.0001).

Among clinical issues related to *S. epidermidis,* biofilm formation on indwelling medical devices and biotic surfaces is frequently cited, which makes therapy more difficult. Thus, biofilm formation assays were performed in the presence of IPM or DltA inhibitor alone or in combination. We conducted these experiments with MRSE 11 that showed robust biofilm formation compared to other tested strains (Fig. S8). When used alone, DltA inhibitor did not decrease biofilm formation, but its interquartile range was multiplied by 4 (Fig. 6). This could reflect a biofilm embrittlement that is revealed during biofilm washing steps. In experiments with IPM alone, the highest sub-MIC IPM concentrations induced a slight increase in biofilm formation with an OD_600_ of nearly 14 units between 0.25 and 16 µg/mL of IPM, compared to 9 units in control conditions (Fig. 6). In experiments with IPM/DltA inhibitor combination, the OD_600_ was decreased by at least 70% with 0.5 mM inhibitor and IPM concentrations from 0.016 µg/mL to 64 µg/mL compared with IPM alone. Although no biofilm eradication was observed even at 1 mM of DltA inhibitor (Fig. S9), intra-biofilm survival was reduced by approximately 1 log when combined with low doses of IPM (from 0.25 to 8 µg/mL).

## DISCUSSION

D-alanylation of TAs in Gram-positive bacteria, mediated by the five proteins DltX, DltA, DltB, DltC and DltD (encoded by the *dltXABCD* operon), turned out to be a promising novel drug target. Although not essential, deficiencies in D-alanylation reduce virulence, biofilm formation, and resistance to antimicrobials (27, 28). Our previous work provided a proof-of-concept for the efficiency of an inhibitor of D-alanylation of TAs in counteracting the antibiotic resistance of enterococci (17) and MRSA (18). Treatment of an *E. faecalis* mutant deficient in D-alanylation with a β-lactam did not modify MICs, but a combination of a DltA inhibitor with a cephalosporin and a very low concentration of ampicillin was highly lethal for several clinical isolates (17). In *S. aureus*, the DltA inhibitor decreased the MICs of several β-lactams of the tested MRSA strains, with the most effective treatment being observed with the combination IPM/DltA inhibitor. Under these conditions, all tested strains showed MICs under the clinical breakpoint of IPM. As *S. epidermidis* is emerging as one of the main causes of HAIs, this study investigated the relevance and effects of using the same DltA inhibitor on this major opportunistic pathogen. The structural comparison of *Se*DltA and *Bsu*DltA revealed two amino acid substitutions in regions previously identified by Yonus et al. (23) as crucial for enzyme activity. However, these are located in the P-loop and hinge regions, which do not interact with the D-alanyl adenylate intermediate. Therefore, we concluded that the DltA inhibitor should be active on *Se*DltA. This is indeed the case since the IC_50_ value of the DltA inhibitor with *Se*DltA protein is in the micromolar range, which is similar to that determined with the *E. faecalis* protein (29).

The combinatorial treatment of representative β-lactams and the DltA inhibitor decreased MICs in most of the tested MRSE clinical isolates. As for *S. aureus*, the most effective treatment was the IPM/inhibitor combination, and more than half of the tested strains showed also significantly reduced MBCs. In contrast to *S. aureus*, four strains (MRSE 5, MRSE 9, MRSE 10, and MRSE 13) were not responsive to this treatment, although fewer strains (*n* = 13) were tested for *S. aureus* than for *S. epidermidis* (*n* = 24). An obvious explanation would be that the DltA inhibitor does not achieve its target because of penetration, stability, or DltA affinity issues in these strains. However, the inhibitor significantly reduces D-alanylation of TAs in all strains used in this study and was highly effective in combination with OXA, decreasing MICs from 256/128 µg/mL to 4 µg/mL in *S. epidermidis* isolates MRSE 5, MRSE 9, and MRSE 13. Surprisingly, antibiotic resistance of some strains was not at all affected in the presence of the inhibitor. This might be due to the presence of housekeeping PBPs with less affinity for β-lactams. A similar reason may explain why two other strains (MRSE 16 and MRSE 20) do not respond to the OXA/inhibitor or FOX/inhibitor combination but for which the IPM/inhibitor combination was highly effective. Understanding why these strains are resistant to the antibiotic/DltA inhibitor combination is expected to shed light on the hitherto unknown molecular mechanism of how inhibition of D-alanylation of TAs sensitizes resistant bacteria to β-lactams.

An interesting new pharmacological effect of the DltA inhibitor revealed for *S. epidermidis* is its anti-virulence effect, since the administration of the DltA inhibitor alone in *G. mellonella* infected with *S. epidermidis* significantly improved the insect survival. This was observed neither for enterococci nor for *S. aureus* (17, 18). We further showed that *S. epidermidis* is able to grow inside the host, independent of the presence of the DltA inhibitor. So, the observed increase in survival of the larvae in the presence of the inhibitor is not due to in-host growth inhibition, but might be linked to the *G. mellonella* immune system. D-alanylated TAs are indeed known to prevent bacterial PG recognition by PGRP-SA (peptidoglycan recognition protein – *S. aureus*), a protein of insects involved in innate immune system mechanisms such as hemolymph clotting, thus allowing the bacterium to evade from it (30). While *S. aureus* exhibits a wide range of virulence factors, the virulence of *S. epidermidis* is mainly explained by its ability to form biofilms. Hussain et al. (31) suggested the role of TAs as adhesins, components involved in the first steps of biofilm formation. Our work suggests that D-alanylation of TAs is crucial in this process for *S. epidermidis* virulence in this model, which is different for *S. aureus*. Since the anti-virulence effect of the DltA inhibitor in *S. epidermidis* is independent of effects of the drug on β-lactam resistance, the molecular mechanisms governing the anti-resistance and anti-virulence effects of the inhibitor are two distinct mechanisms. However, definitively attributing reduced MRSE virulence in the *G. mellonella* model to DltA inhibition requires validation with an *S. epidermidis dltA* mutant. Unfortunately, all attempts to construct this mutant in the clinical strains used in this study have failed so far.

In conclusion, the DltA inhibitor tested in this study has interesting effects on methicillin-resistant clinical strains of *S. epidermidis*. It reduces β-lactam resistance, virulence, and biofilm formation. The next steps will focus on deciphering the molecular mechanisms behind these actions of the DltA inhibitor. It is expected that this will shed light, especially on the hitherto unknown virulence factors of this emerging pathogen.

## SUPPLEMENTAL MATERIAL

Supplemental material aac.01822-24-s0001.docx Figures S1 to S9; Tables S1 to S4.

ASM does not own the copyrights to Supplemental Material that may be linked to, or accessed through, an article. The authors have granted ASM a non-exclusive, world-wide license to publish the Supplemental Material files. Please contact the corresponding author directly for reuse.
