# Supplementary material for: D-alanylation of lipoteichoic acids inhibitor provides anti-virulence and anti-resistance effects against methicillin-resistant Staphylococcus epidermidis
Source: Antimicrob Agents Chemother. 2025 Mar 21;69(5):e01822-24. doi: 10.1128/aac.01822-24 (PMC12057345; doi:10.1128/aac.01822-24)
Supplement: Supplemental material — Figures S1 to S9; Tables S1 to S4. [file aac.01822-24-s0001.docx]

**
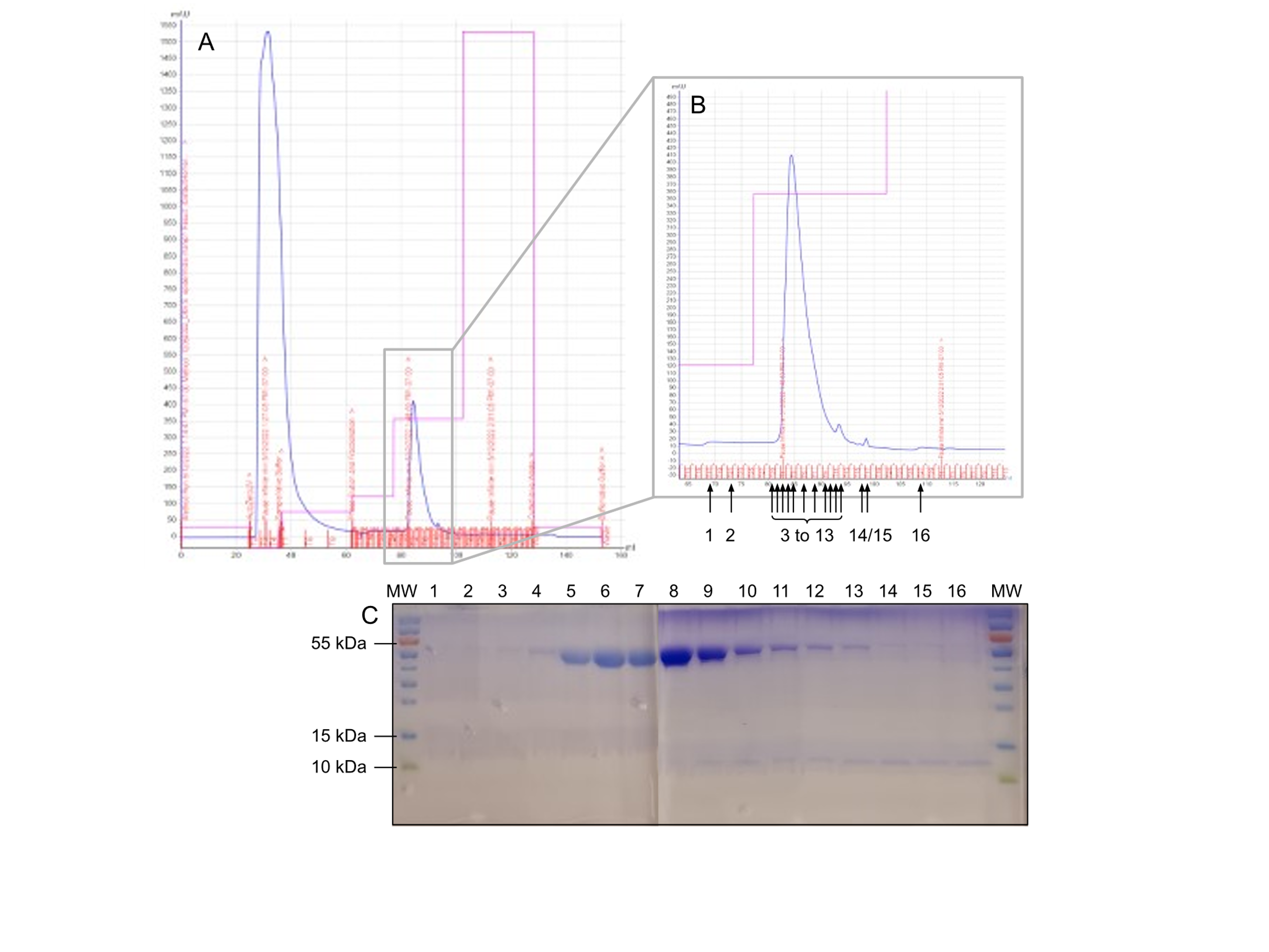
**

**Figure S1: Over-expression and purification of DltA.** Chromatography was performed using a 5-mL HisTrap^TM^ FF Crude set up on a ÄKTA Start system. Pink and blue lines represent the gradient of imidazole and UV-absorbance, respectively. (A) Impurities were eluted with 15 mL (2.5 mL/min) of Tp containing 50 mM of imidazole, then (B) the protein of interest was eluted with a gradient of 65 mL of imidazole from 60 to 500 mM (2.5 mL/min). Numbered arrows correspond to several fractions of elution that were (C) loaded on SDS-PAGE. MW: molecular weight.


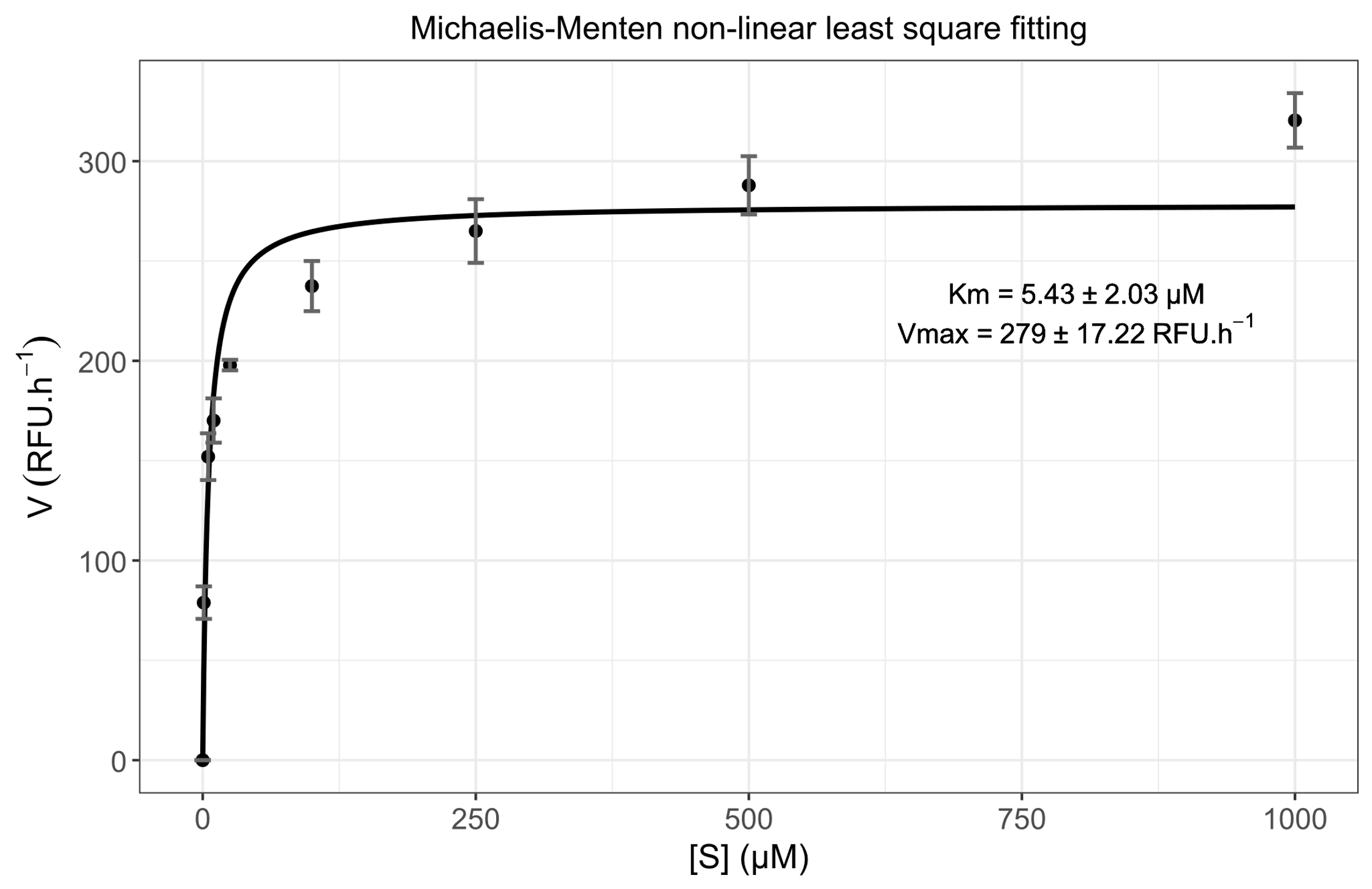


**Figure S2: Determination of *Se*DltA enzymatic parameters.** The initial reaction rate was determined for a wide range of D-alanine concentrations (from 0 to 1000 µM) in the presence of 1.5 µM of pre-treated DltA and 20 µM of ATP. The data were then fitted to the Michaelis-Menten equation using an R script (v4.3.3; R Core Team, 2021; https://www.R-project.org/) adapted from the *renz* R package (Aledo, 2022)(23). Black dots and grey lines correspond to the mean and the 95% confidence interval of four replicates.


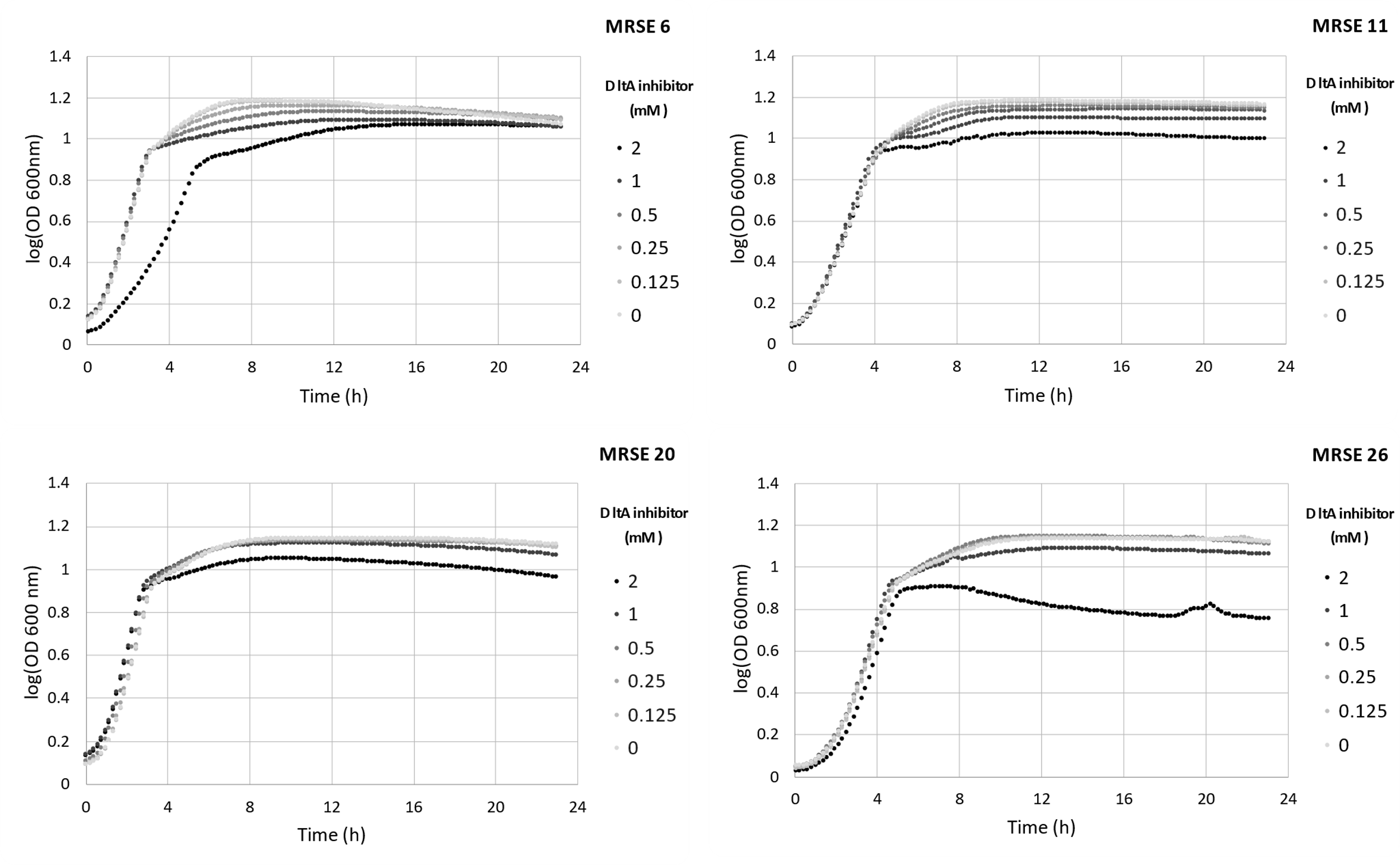
**Figure S3: Growth kinetics of several clinical isolates of *S. epidermidis*.** Four clinical MRSE strains were grown in BHI medium at 37°C for 24 h with increasing DltA inhibitor concentrations. Optical density at 600 nm was measured every 10 min after a brief shaking. Each dot represents the mean value of two biological replicates containing two technical replicates.

**
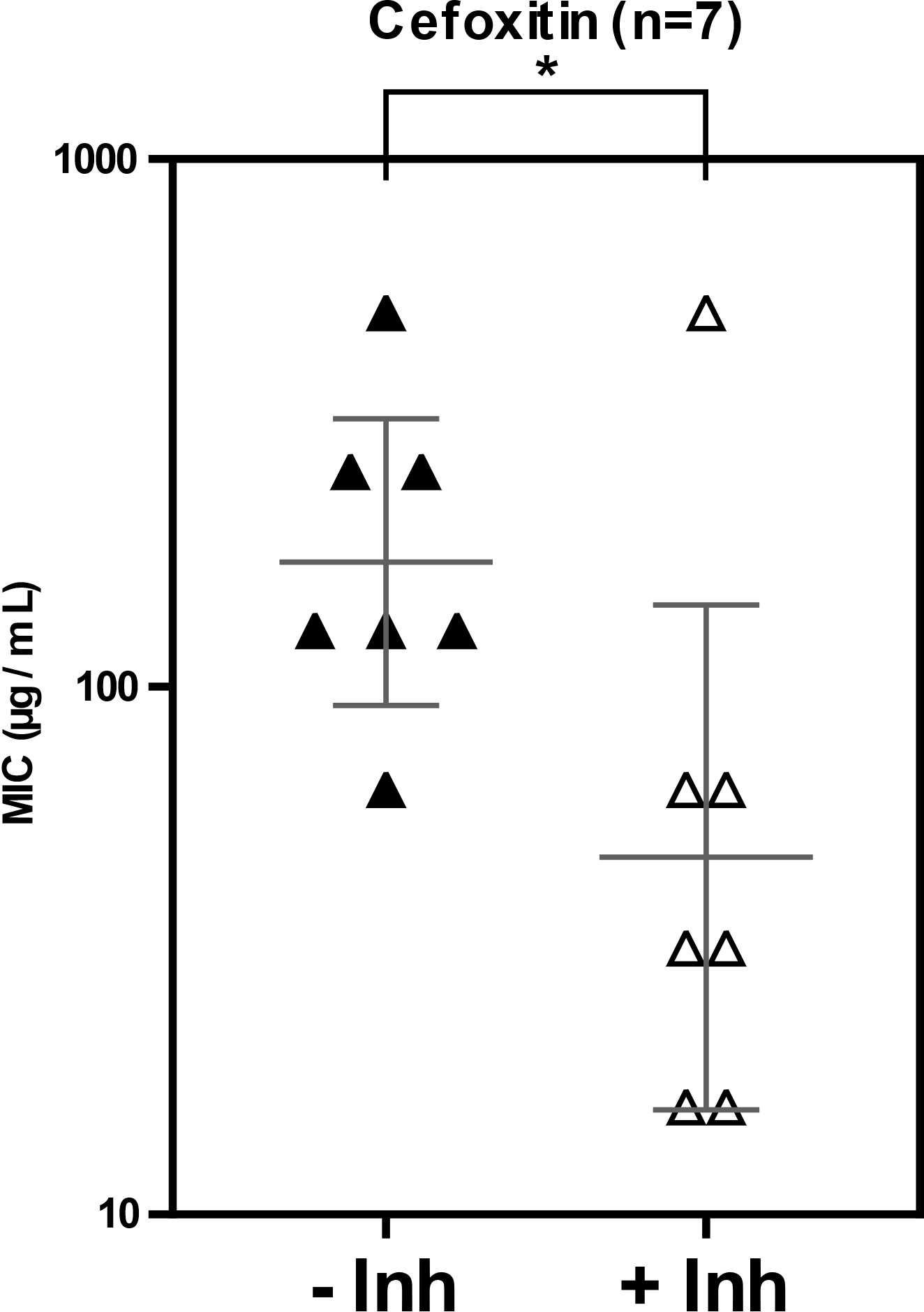
**

**Figure S4: Effect of DltA inhibitor on MICs of cefoxitin against MRSE-clinical isolates.** MICs were assessed after 24 h of incubation in BHI medium in absence (full) or presence (empty) of DltA inhibitor (Inh) at the concentration of 1 mM. Each dot represents the most frequently observed MIC among at least three biological replicates per clinical isolate. N-values (n) give the number of strains assessed for each antibiotic. Gray bars correspond to the geometric mean and the 95% confidence interval. *: P-value <0,05 (one-tailed Wilcoxon matched-pairs signed rank test, realized on the “GraphPad Prism 8.0.1” software (Boston, Massachusetts USA, www.graphpad.com)).

**
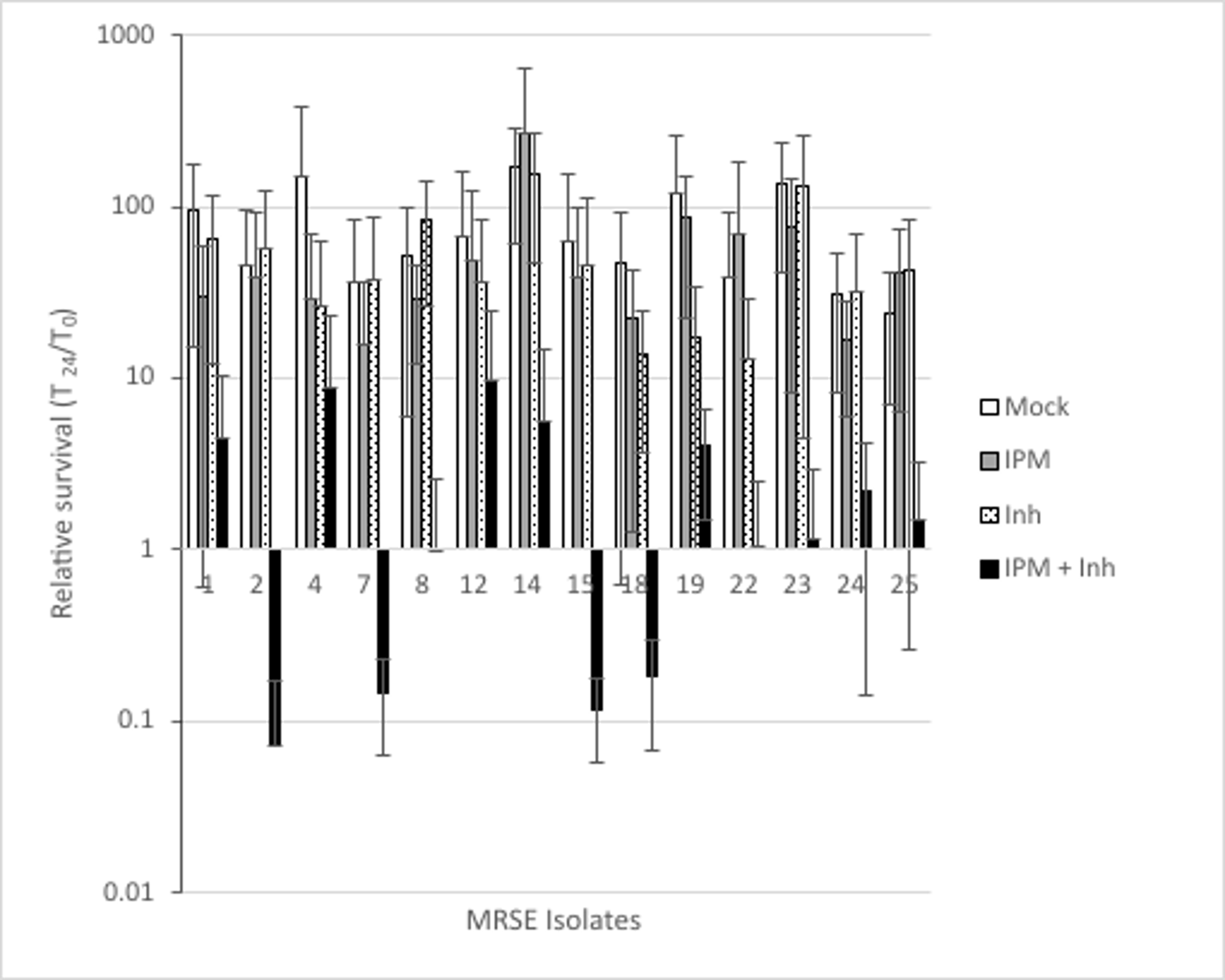
**

**Figure S5: Relative survivals of MRSE clinical isolates in presence of IPM/DltA inhibitor combination reveal a bacteriostatic effect.** Early-log phase cultures of *S. epidermidis* were incubated at 37°C at time 0 (T_0_) in absence (white) or presence of 1 µg/mL of IPM (grey), 1 mM of DltA inhibitor (dotted white) or both (black). After 24 h (T_24_), the survival was determined by plate counting and the relative survival (T_24_/T_0_) was calculated. Results are represented as the mean ± SD survival from three biological replicates.


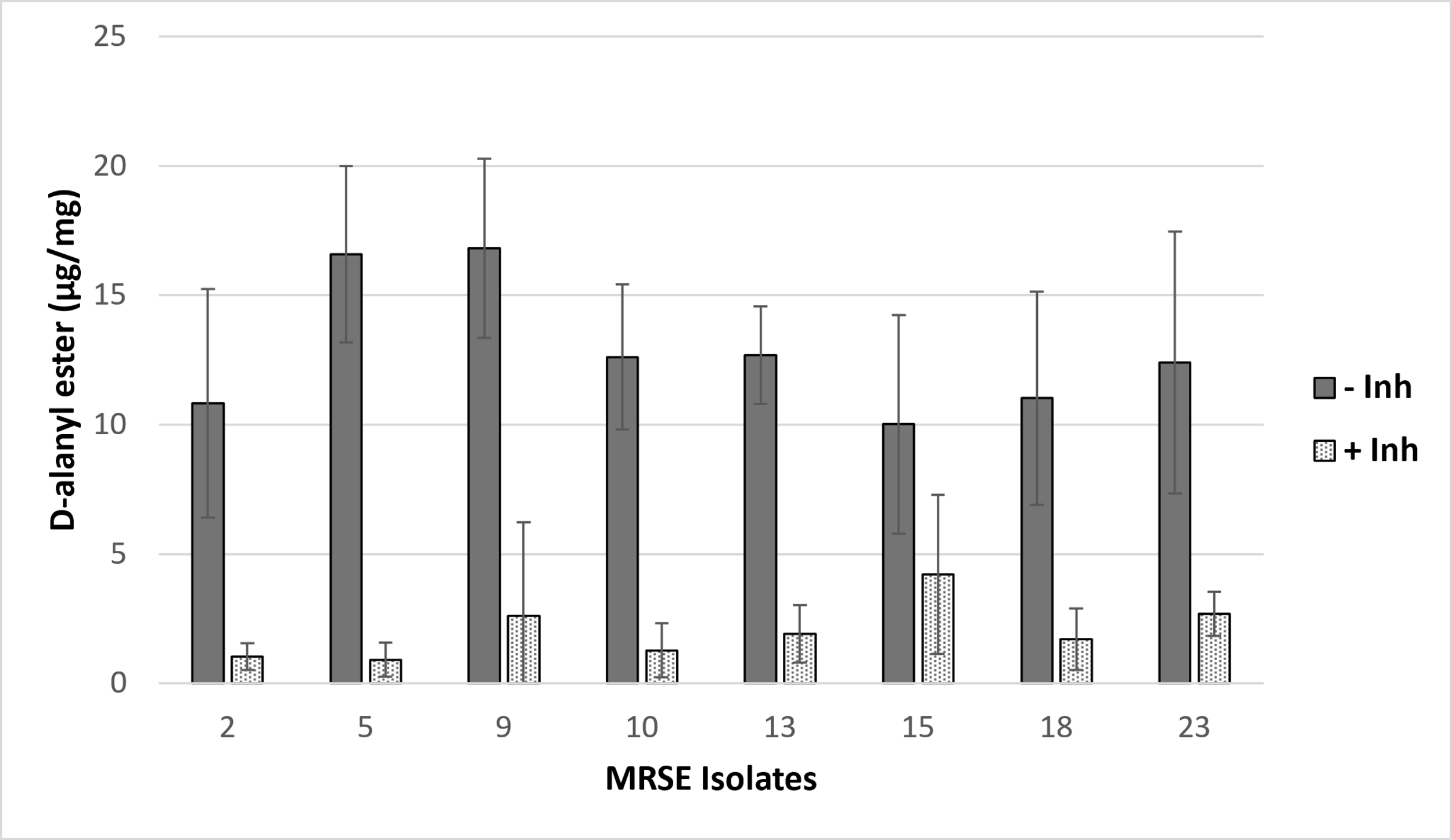


**Figure S6: DltA inhibitor inhibits wall D-alanylation of *S. epidermidis***. Several MRSE strains were grown in BHI for 16 h with or without DltA inhitor (1 mM). Data are represented as the mean ± SD µg of D-alanyl ester per mg of cell wall. At least two biological replicates each containing two technical replicates were assessed.


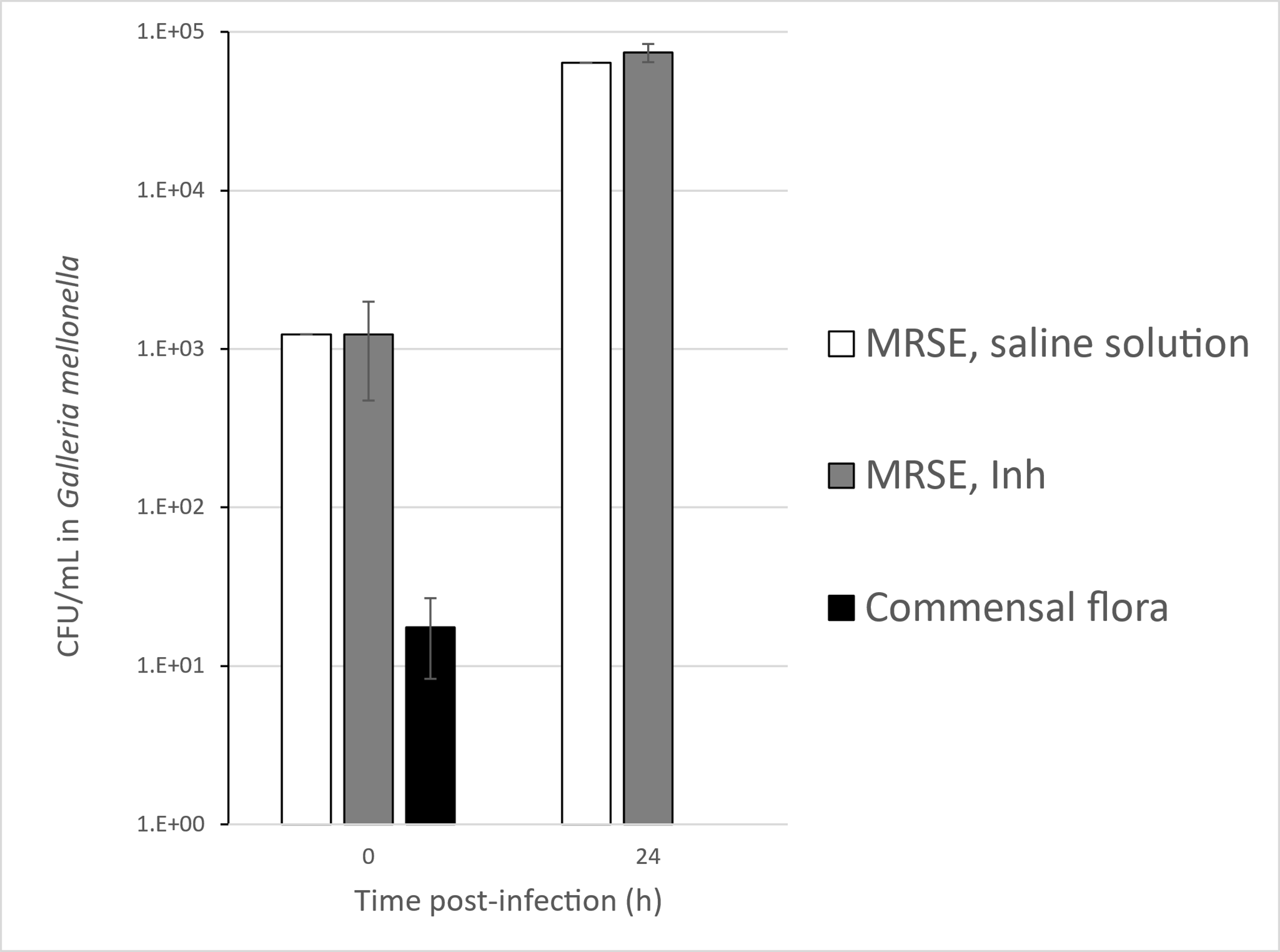


**Figure S7: Colonization assay of MRSE 11 in *G. mellonella.*** Six-week-old *G. mellonella* larvae were infected at time 0 (T_0_) by 10^3^ CFU of MRSE 11 and treated 2 h post-infection with saline solution (white) or DltA inhibitor (grey) (48.5 mg/kg). In host bacterial load at T_0_ and T_24h_ was assessed by plating the crushed larvae on BHI agar containing erythromycin at a concentration of 75 µg/mL. The black bar corresponds to the commensal flora. Results are represented as the mean ± SD survival from three biological replicates.


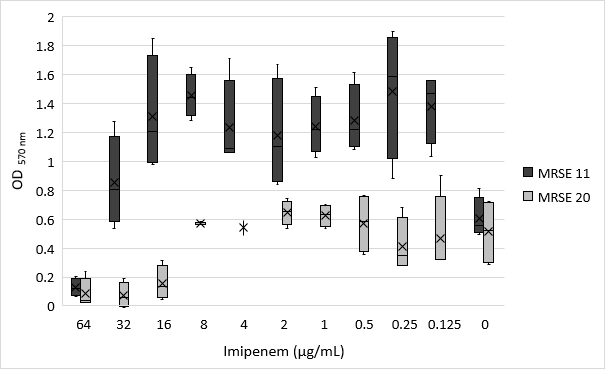


**Figure S8: Preliminary tests for Biofilm formation of MRSE 11 and MRSE 20 isolates assessment.** Biofilm formation was assessed on bacteria grown in BHI supplemented with 2% glucose for 24 h. Data are represented in box plots, providing the distribution of four technical replicates as well as the means (cross), medians (horizontal bars) and outliers (circles). The values are corrected by subtracting the mean of negative controls (medium only).


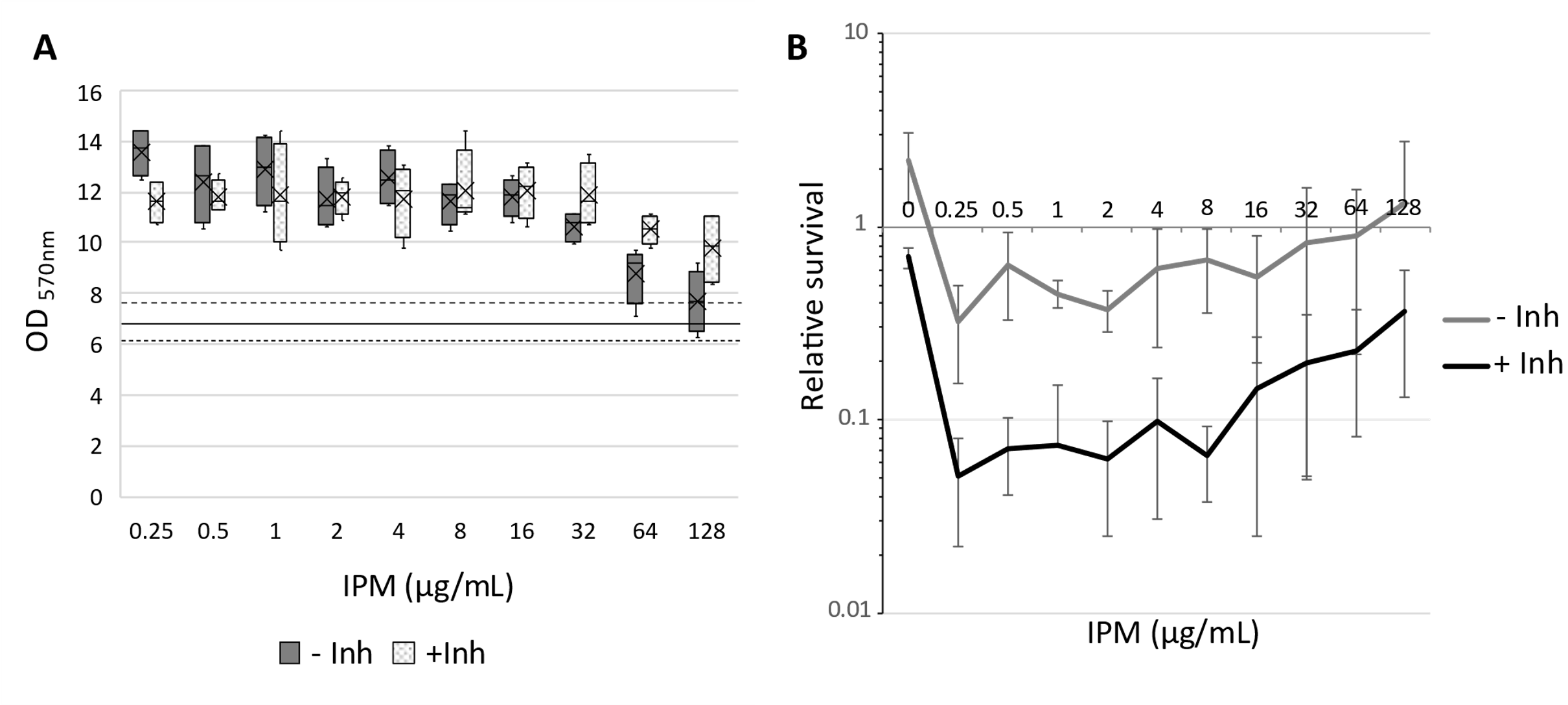


**Figure S9: Biofilm eradication assay.** Biofilm formation was performed in BHI broth supplemented with 2% glucose at 37°C for 24h. Biofilm was then washed and treated by BHI broth supplemented with 2% glucose, IPM with (dotted) or without (grey) DLT-1 (Inh, 1mM). (A) At 48h, biofilm was quantified. Data are represented in box plots, providing the distribution of four technical replicates as well as the means (cross) and medians (horizontal bars). The values are corrected by subtracting the mean of negative controls (medium only). Black line and dotted black lines represent the mean ± SD value obtained at T_24_. (B) At 24h (T_24_) and 48h (T_48_), viable intra-biofilm cells where counted. Related survival (T_48_/T_24_) are represented as the mean ± SD of three biological replicates containing each four technical replicates.

**Table S1: Primers used in this study.**

| **Primer** | **Sequence (5’-3’)** | **Use** |
| --- | --- | --- |
| OCG232 | CACCACCACCACCACCAC | Pet29b(+) amplification |
| OCG233 | CATATGTATATCTCCTTCTTAAAGTTAAACAAA | Pet29b(+) amplification |
| F_SE_pET*dltA* | GTTTAACTTTAAGAAGGAGATATA**CATATG**GCAGACTTAATTAATATTCT | DltA over-expression, ***Nde*I**, START |
| R_SE_pET*dltA* | AGCCGGATCTCA*GTGGTGGTGGTGGTGGTG*TCCGTTTACAACCTCGGCA | DltA over-expression, *6xHis-Tag*, STOP |
| F_SE_pET*dltC* | TTAACTTTAAGAAGGAGATATA**CATATG**GAATTTAGAGAACAAGTATTAG | DltC over-expression, ***Nde*I**, START |
| R_SE_pET*dltC* | GCCGGATCTCA*GTGGTGGTGGTGGTGGTG*TCGTAACTCTTCTAAAGCTTC | DltC over-expression, *6xHis-Tag*, STOP |
| OCG240 | CGCGAAATTAATACGACTCACTATA | Upstream of Pet29b(+) MCS |
| OCG241 | GGGGTTATGCTAGTTATTGCTCA | Downstream of Pet29b(+) MCS |

**Table S2: Buffers composition.**

|  | **Compounds** | **Supplier / Reference** |
| --- | --- | --- |
| **Transformation and storage solution (TSS)** | PEG 3000 (100 g/L) | Merck (Darmstadt, Germany) |
|  | MgCl_2_ (1 M) | Merck (Darmstadt, Germany) |
|  | DMSO (5%) | Honeywell (Charlotte, USA) |
|  | LB broth | (Bertani, 1951) |
| **KCM 5X** | KCl (2M) | Sigma-Aldrich (Saint-Louis, USA) |
|  | MgCl_2_ (1 Mm) | Merck (Darmstadt, Germany) |
|  | CaCl_2_ (1M) | Merck (Darmstadt, Germany) |
| **Purification buffer (Tp)** | Na_2_HPO_4_ /NaH_2_PO_4_, Ph8 (50Mm) | Sigma-Aldrich (Saint-Louis, USA) / VWR (Radnor, USA) |
|  | NaCl (300 Mm) | Honeywell (Charlotte, USA) |
|  | Imidazole (variable) | Sigma-Aldrich (Saint-Louis, USA) |
| **Desalination buffer (EB-3)** | Tris-HCl (100 Mm) | fisher bioreagents (Illkirch-Graffenstaden, France) |
|  | MgCl_2_ (1 Mm) | Merck (Darmstadt, Germany) |
|  | Glycerol (15%) | ITW Reagents |

**Table S3:** **Effect of DltA inhibitor on MICs and MBCs of various β-lactams against a panel of MRSE clinical isolates.**

|  | Oxacillin | | | | Cefoxitin | | | | Imipenem | | | |
| --- | --- | --- | --- | --- | --- | --- | --- | --- | --- | --- | --- | --- |
|  | **MIC** | | MBC | | **MIC** | | MBC | | **MIC** | | MBC | |
| **Strain** | **- Inh** | **+ Inh** | **- Inh** | **+ Inh** | **- Inh** | **+ Inh** | **- Inh** | **+ Inh** | **- Inh** | **+ Inh** | **- Inh** | **+ Inh** |
| **MRSE1** | **16** | **2** | 256 | 32 | **64** | N.D. | 64 | N.D. | **32** | **0.25** | 64 | 0.5 |
| **MRSE 2** | **<2** | **0.5** | 16 | 1 | **16** | N.D. | 64 | N.D. | **8** | **≤0.0625** | 16 | 0.125 |
| **MRSE 4** | **16** | **1** | 32 | 16 | **64** | N.D. | 128 | N.D. | **8** | **0.125** | 32 | 2 |
| **MRSE 5** | **128** | **4** | 256 | 64 | **128** | N.D. | 256 | N.D. | **64** | **64** | 128 | 128 |
| **MRSE 6** | **256** | **2** | 256 | 16 | **256** | **32** | >256 | N.D. | **64** | **0.25** | 64 | 32 |
| **MRSE 7** | **32** | **2** | 64 | 2 | **32** | N.D. | 64 | N.D. | **16** | **0.125** | 32 | 0.5 |
| **MRSE 8** | **16** | **2** | 16 | 8 | **32** | N.D. | 128 | N.D. | **4** | **0.0625** | 16 | 0.25 |
| **MRSE 9** | **128** | **4** | 256 | 64 | **128** | N.D. | 256 | N.D. | **64** | **32** | 128 | 128 |
| **MRSE 10** | **512** | **64** | 512 | 128 | **512** | **512** | 512 | N.D. | **128** | **64** | 128 | 128 |
| **MRSE 11** | **256** | **2** | 256 | 64 | **256** | **64** | 256 | N.D. | **64** | **0.5** | 64 | 16 |
| **MRSE 12** | **128** | **4** | 128 | 4 | **64** | N.D. | 256 | N.D. | **32** | **0.25** | 32 | 2 |
| **MRSE 13** | **256** | **4** | 512 | 32 | **128** | N.D. | 256 | N.D. | **64** | **32** | 128 | 128 |
| **MRSE 14** | **64** | **2** | 128 | 64 | **64** | N.D. | 128 | N.D. | **8** | **0.0625** | 32 | 1 |
| **MRSE 15** | **16** | **1** | 64 | 4 | **128** | **16** | 256 | N.D. | **32** | **0.25** | 64 | 2 |
| **MRSE 16** | **256** | **256** | 512 | 512 | **128** | **64** | 256 | N.D. | **64** | **0.5** | 128 | 32 |
| **MRSE 18** | **8** | **1** | 8 | 2 | **32** | N.D. | N.D. | N.D. | **32** | **≤0.125** | 32 | 1 |
| **MRSE 19** | **16** | **1** | 16 | 2 | **32** | N.D. | N.D. | N.D. | **16** | **0.25** | 32 | 2 |
| **MRSE 20** | **128** | **32** | 128 | 128 | **64** | **16** | 128 | 32 | **8** | **0.25** | 16 | 0.5 |
| **MRSE 21** | **64** | **4** | 128 | 4 | **32** | N.D. | 64 | N.D. | **32** | **4** | 32 | 4 |
| **MRSE 22** | **128** | **1** | 64 | 4 | **64** | N.D. | N.D. | N.D. | **8** | **0.0625** | 32 | 4 |
| **MRSE 23** | **32** | **1** | 64 | 1 | **64** | N.D. | >256 | N.D. | **8** | **0.0625** | 32 | 0.5 |
| **MRSE 24** | **8** | **1** | 64 | 2 | **64** | N.D. | 256 | N.D. | **32** | **0.25** | 32 | 2 |
| **MRSE 25** | **512** | **32** | 256 | 32 | **64** | N.D. | 256 | N.D. | **16** | **0.125** | 32 | 0.5 |
| **MRSE 26** | **256** | **2** | 256 | 16 | **128** | **32** | 128 | 32 | **64** | **0.25** | 64 | 32 |
| MSSE 1 | **<0.5** | N.D. | <0.5 | N.D. | **4** | N.D. | N.D. | N.D. | **<0.5** | N.D. | <0.5 | N.D. |

**Table S4: Effect of various DltA inhibitor concentrations on MIC of IPM (µg/mL) against 4 clinical isolates of *S. epidermidis*.**

|  | **[DLT-1] (mM)** | **0** | **0.1** | **0.25** | **0.5** | **0.75** | **1** |
| --- | --- | --- | --- | --- | --- | --- | --- |
| **Imipenem** | **MRSE 6** | 32 | 32 | 16 | 2 | 1 | 0.25 |
|  | **MRSE 11** | 64 | 32 | 32 | 8 | 1 | 0.25 |
|  | **MRSE 20** | 16 | 16 | 2 | 0.5 | 0.25 | 0.25 |
|  | **MRSE 26** | 64 | 32 | 32 | 32 | 16 | 0.25 |
